# Draft Genome Sequence of the *Wolbachia* Endosymbiont of *Drosophila suzukii*

**DOI:** 10.1128/genomeA.00032-13

**Published:** 2013-02-28

**Authors:** Stefanos Siozios, Alessandro Cestaro, Rupinder Kaur, Ilaria Pertot, Omar Rota-Stabelli, Gianfranco Anfora

**Affiliations:** Research and Innovation Centre, Fondazione Edmund Mach, San Michele all’Adige (TN), Italy

## Abstract

*Wolbachia* is one of the most successful and abundant symbiotic bacteria in nature, infecting more than 40% of the terrestrial arthropod species. Here we report the draft genome sequence of a novel *Wolbachia* strain named “*w*Suzi” that was retrieved from the genome sequencing of its host, the invasive pest *Drosophila suzukii*.

## GENOME ANNOUNCEMENT

*Drosophila suzukii* (Matsumura) (*Diptera Drosophilidae*) is an invasive and destructive crop pest native to Southeast Asia that recently invaded Western countries, threatening both European and American fruit production (1, 2). The exploitation of symbiotic microorganisms for insect pest control has received considerable interest over the last few years (3). Owing to a pandemic distribution and unique ability to manipulate host reproduction, *Wolbachia* symbiotic bacteria are considered to be great candidates for their biocontrol potential (4, 5). Advances in genomics provide new opportunities for improving our understanding of *Wolbachia* biology.

As a serendipitous byproduct of *D. suzukii* genome sequencing (L. Ometto, A. Cestaro, S. Ramasamy, A. Grassi, S. Revadi, S. Siozios, M. Moretto, P. Fontana, C. Varotto, D. Pisani, T. Dekker, N. Wrobel, R. Viola, I. Pertot, D. Cavalieri, M. Blaxter, G. Anfora, and O. Rota-Stabelli, submitted for publication) the genome of its *Wolbachia* endosymbiont (“*w*Suzi”) was sequenced. By using the genome sequences of the *w*Mel, *w*Ri, *w*Ana, wWil, and *w*Sim *Wolbachia* strains as probes, we searched the raw sequences from *D. suzukii* sequencing and retrieved 1,082,694 *Wolbachia* matched reads (IlluminaHiseqII 100-bp reads). Among the 1,082,694 reads, 1,054,920 were paired, corresponding to two pair-end libraries with average insert sizes of 180 and 300 bp, while the remaining 27,774 reads were singletons. *De novo* assembly was performed using the packages MIRA (6) under default parameters and Velvet (7) using a kmer of 65; we further assisted assembly using the AMOS_cmp assembler (8) and the genome of the *w*Ri strain infecting *D. simulans* as a reference (9). To identify ambiguously assembled contigs, the three assemblies were simultaneously mapped against the *w*Ri genome using the Geneious mapping algorithm (10). The final assembly yielded approximately 1.35 Mbp in 110 contigs, with a maximum length of 89,713 bp, a mean of 12,272 bp, and an average depth of coverage of 60×. The genome has an average G+C content of 35.2% and contains approximately 1,262 open reading frames (ORFs), as predicted by Glimmer v3.02 (11), and one copy of the 16S, 23S, and 5S rRNA genes and 34 tRNA genes, as predicted by tRNAscan_SE (12).

The draft genome of *w*Suzi displays great similarity with the *w*Ri genome and covers approximately 98% of its length. Indeed, all of the markers commonly used to discriminate between different *Wolbachia* strains (13–17) revealed no substitutions between *w*Ri and *w*Suzi. The only exception was *dnaA*, with only one nonsynonymous substitution separating the two strains. However, we were able to identify and validate with PCR several structural variations, such as indels and genomic rearrangements. The most relevant deletion is of approximately 21 kbp and is upstream of the WORiB prophage; this region includes mainly ankyrin repeat genes. There are also two large-scale rearrangements distinguishing the two genomes. The largest one corresponds to an inversion of a segment at least 80 kbp long. Finally, we found that most of the polymorphism between the two strains involves transposable elements: we could detect approximately 34 deletions of insertion sequence (IS) elements in *w*Suzi compared to the *w*Ri genome. Our data indicate that *D. suzukii* carries a novel strain of *Wolbachia* whose close relationship with *w*Ri hampers its discrimination with the classical MLST approach (16, 17). Further genetic and comparative studies will provide new insights into the biology of this *Drosophila-Wolbachia* association.

### Nucleotide sequence accession numbers.

The genome sequence of the *Wolbachia* endosymbiont of *D. suzukii* has been deposited at EMBL under accession numbers CAOU02000001 to CAOU02000110. The sequences are also available from the website of Fondazione Edmund Mach (http://genomics.research.iasma.it/ds/twiki/bin/view/Main/ScaricaGenomi).
